# Early Identification of Autism Spectrum Disorder Among Children Aged 4 Years — Autism and Developmental Disabilities Monitoring Network, 11 Sites, United States, 2020

**DOI:** 10.15585/mmwr.ss7201a1

**Published:** 2023-03-24

**Authors:** Kelly A. Shaw, Deborah A. Bilder, Dedria McArthur, Ashley Robinson Williams, Esther Amoakohene, Amanda V. Bakian, Maureen S. Durkin, Robert T. Fitzgerald, Sarah M. Furnier, Michelle M. Hughes, Elise T. Pas, Angelica Salinas, Zachary Warren, Susan Williams, Amy Esler, Andrea Grzybowski, Christine M. Ladd-Acosta, Mary Patrick, Walter Zahorodny, Katie K. Green, Jennifer Hall-Lande, Maya Lopez, Kristen Clancy Mancilla, Ruby H.N. Nguyen, Karen Pierce, Yvette D. Schwenk, Josephine Shenouda, Kate Sidwell, Alison Vehorn, Monica DiRienzo, Johanna Gutierrez, Libby Hallas, Allison Hudson, Margaret H. Spivey, Sydney Pettygrove, Anita Washington, Matthew J. Maenner

**Affiliations:** ^1^National Center on Birth Defects and Developmental Disabilities, CDC, Atlanta, Georgia; ^2^University of Utah School of Medicine, Salt Lake City, Utah; ^3^Oak Ridge Institute for Research and Education, Oak Ridge, Tennessee; ^4^University of Wisconsin, Madison, Wisconsin; ^5^Washington University, St. Louis, Missouri; ^6^Johns Hopkins Bloomberg School of Public Health, Baltimore, Maryland; ^7^Vanderbilt University Medical Center, Nashville, Tennessee; ^8^University of Minnesota, Minneapolis, Minnesota; ^9^University of California, San Diego, California; ^10^Rutgers New Jersey Medical School, Newark, New Jersey; ^11^University of Arkansas for Medical Sciences, Little Rock, Arkansas; ^12^University of Arizona, Tucson, Arizona

## Abstract

**Problem/Condition:**

Autism spectrum disorder (ASD).

**Period Covered:**

2020.

**Description of System:**

The Autism and Developmental Disabilities Monitoring Network is an active surveillance program that estimates prevalence and characteristics of ASD and monitors timing of ASD identification among children aged 4 and 8 years. In 2020, a total of 11 sites (located in Arizona, Arkansas, California, Georgia, Maryland, Minnesota, Missouri, New Jersey, Tennessee, Utah, and Wisconsin) conducted surveillance of ASD among children aged 4 and 8 years and suspected ASD among children aged 4 years. Surveillance included children who lived in the surveillance area at any time during 2020. Children were classified as having ASD if they ever received 1) an ASD diagnostic statement in an evaluation, 2) a special education classification of autism (eligibility), or 3) an ASD *International Classification of Diseases* (ICD) code (revisions 9 or 10). Children aged 4 years were classified as having suspected ASD if they did not meet the case definition for ASD but had a documented qualified professional’s statement indicating a suspicion of ASD. This report focuses on children aged 4 years in 2020 compared with children aged 8 years in 2020.

**Results:**

For 2020, ASD prevalence among children aged 4 years varied across sites, from 12.7 per 1,000 children in Utah to 46.4 in California. The overall prevalence was 21.5 and was higher among boys than girls at every site. Compared with non-Hispanic White children, ASD prevalence was 1.8 times as high among Hispanic, 1.6 times as high among non-Hispanic Black, 1.4 times as high among Asian or Pacific Islander, and 1.2 times as high among multiracial children. Among the 58.3% of children aged 4 years with ASD and information on intellectual ability, 48.5% had an IQ score of ≤70 on their most recent IQ test or an examiner’s statement of intellectual disability. Among children with a documented developmental evaluation, 78.0% were evaluated by age 36 months. Children aged 4 years had a higher cumulative incidence of ASD diagnosis or eligibility by age 48 months compared with children aged 8 years at all sites; risk ratios ranged from 1.3 in New Jersey and Utah to 2.0 in Tennessee. In the 6 months before the March 2020 COVID-19 pandemic declaration by the World Health Organization, there were 1,593 more evaluations and 1.89 more ASD identifications per 1,000 children aged 4 years than children aged 8 years received 4 years earlier. After the COVID-19 pandemic declaration, this pattern reversed: in the 6 months after pandemic onset, there were 217 fewer evaluations and 0.26 fewer identifications per 1,000 children aged 4 years than children aged 8 years received 4 years earlier. Patterns of evaluation and identification varied among sites, but there was not recovery to pre-COVID-19 pandemic levels by the end of 2020 at most sites or overall. For 2020, prevalence of suspected ASD ranged from 0.5 (California) to 10.4 (Arkansas) per 1,000 children aged 4 years, with an increase from 2018 at five sites (Arizona, Arkansas, Maryland, New Jersey, and Utah). Demographic and cognitive characteristics of children aged 4 years with suspected ASD were similar to children aged 4 years with ASD.

**Interpretation:**

A wide range of prevalence of ASD by age 4 years was observed, suggesting differences in early ASD identification practices among communities. At all sites, cumulative incidence of ASD by age 48 months among children aged 4 years was higher compared with children aged 8 years in 2020, indicating improvements in early identification of ASD. Higher numbers of evaluations and rates of identification were evident among children aged 4 years until the COVID-19 pandemic onset in 2020. Sustained lower levels of ASD evaluations and identification seen at a majority of sites after the pandemic onset could indicate disruptions in typical practices in evaluations and identification for health service providers and schools through the end of 2020. Sites with more recovery could indicate successful strategies to mitigate service interruption, such as pivoting to telehealth approaches for evaluation.

**Public Health Action:**

From 2016 through February of 2020, ASD evaluation and identification among the cohort of children aged 4 years was outpacing ASD evaluation and identification 4 years earlier (from 2012 until March 2016) among the cohort of children aged 8 years in 2020 . From 2016 to March 2020, ASD evaluation and identification among the cohort of children aged 4 years was outpacing that among children aged 8 years in 2020 from 2012 until March 2016. The disruptions in evaluation that coincided with the start of the COVID-19 pandemic and the increase in prevalence of suspected ASD in 2020 could have led to delays in ASD identification and interventions. Communities could evaluate the impact of these disruptions as children in affected cohorts age and consider strategies to mitigate service disruptions caused by future public health emergencies.

## Introduction

Autism spectrum disorder (ASD) is a developmental disability characterized by deficits in social interaction or communication and the presence of restricted interests or repetitive behaviors. The American Academy of Pediatrics recommends that pediatric care providers screen all children for ASD at ages 18 and 24 months in addition to regular developmental surveillance (*1*). Early developmental screening and receipt of services for children with ASD also are core national objectives for *Healthy People 2030* (*2*). During early childhood, ongoing neurodevelopmental processes present the opportunity to optimize children’s ability to develop language and social skills (*3*–*5*). Early ASD identification is important to ensure children have access to services they might need to support development of these skills.

The Autism and Developmental Disabilities Monitoring (ADDM) Network has reported biennial ASD estimates since 2000 and began tracking ASD identification among children aged 4 years in a subset of sites in 2010 (*6*). In 2018, surveillance among this age group expanded to the full ADDM Network. New patterns in ASD prevalence by race and ethnicity emerged, with children from groups with historically lower prevalence, including non-Hispanic Black and Hispanic children, and children in lower socioeconomic status (SES) neighborhoods having the highest prevalence (*7*). Since 2016, comparisons with children aged 8 years have shown more early identification of ASD by age 48 months among younger cohorts (*7*,*8*).

The 2020 surveillance year includes the COVID-19 pandemic declaration by the World Health Organization in March 2020 (https://www.who.int/europe/emergencies/situations/covid-19) and ensuing shutdowns in the United States. The Act Early Response to COVID-19 project performed a rapid needs assessment in 2020, surveying key partners from early childhood programs and systems in 43 U.S. states and territories. Overall, 91% of the 349 participants reported the COVID-19 pandemic had “highly impacted” early identification of children with developmental delays and disabilities (*9*). Forty-eight percent of these participants reported during fall 2020 that the number of children served by early childhood programs and systems had decreased since the start of the pandemic. The ADDM Network is uniquely positioned to provide population-based measures that can show disruptions to timely evaluation and identification of ASD.

This report provides data on early ASD identification among children aged 4 years in 11 U.S. communities, including prevalence and characteristics of children with ASD and suspected ASD in 2020, and comparisons with children aged 8 years to show patterns in identification and emergence of possible impacts of the COVID-19 pandemic. These data can be used for ongoing monitoring of trends and to support efforts to ensure early and equitable identification of children with ASD.

## Methods

### Surveillance Sites and Procedures

ADDM Network sites conducted surveillance of ASD among children aged 4 and 8 years and suspected ASD among children aged 4 years in 2020 in defined areas of Arizona, Arkansas, California, Georgia, Maryland, Minnesota, Missouri, New Jersey, Tennessee, Utah, and Wisconsin (Table 1). All sites functioned as public health authorities under the Health Insurance Portability and Accountability Act of 1996 Privacy Rule and met applicable local institutional review board, privacy, and confidentiality requirements under 45 CFR 46.

**TABLE 1:** Prevalence* of autism spectrum disorder among children aged 4 years and percentage of children who had an autism spectrum disorder diagnosis, special education eligibility, or an International Classification of Diseases code — Autism and Developmental Disabilities Monitoring Network, 11 sites, 2020

Site	Surveillance area description	Denominator	No. aged 4 yrs with ASD	ASD prevalence (95% CI)^†^	% Who had an ASD diagnostic statement	% Who had ASD special education eligibility	% Who had ASD ICD code
Arizona	Part of one county in metropolitan Phoenix	13,349^§^	209	15.7 (13.7–17.9)	75.6	11.0	63.2
Arkansas	21 counties in central Arkansas	15,150	245	16.2 (14.3–18.3)	95.5	36.3	84.5
California	Part of one county in metropolitan San Diego	16,719^§^	776	46.4 (43.3–49.7)	85.8	69.7	88.3
Georgia	Two counties in metropolitan Atlanta	21,985	384	17.5 (15.8–19.3)	77.3	41.1	71.1
Maryland	Five counties in suburban Baltimore	20,745	352	17.0 (15.3–18.8)	90.3	43.2	72.4
Minnesota	Parts of three counties in the Twin Cities metropolitan area	16,326^§^	305	18.7 (16.7–20.9)	54.1	75.1	22.0
Missouri	Five counties in metropolitan St. Louis	24,476	456	18.6 (17.0–20.4)	95.0	17.5	92.5
New Jersey	Two counties in the New York metropolitan area	19,120	473	24.7 (22.6–27.0)	98.3	9.3	76.7
Tennessee	11 counties in middle Tennessee	26,474	737	27.8 (25.9–29.9)	71.0	32.8	92.7
Utah	Three counties in northern Utah	24,330	308	12.7 (11.3–14.1)	79.9	25.0	81.8
Wisconsin	Eight counties in southeastern Wisconsin	28,852	651	22.6 (20.9–24.3)	65.1	35.3	84.0
**Total**		**227,526**	**4,896**	**21.5 (20.9**–**22.1)**	**80.2**	**38.1**	**79.4**

**Abbreviations:** ASD = autism spectrum disorder; ICD = *International Classification of Diseases*.

* Per 1,000 children aged 4 years.

^†^ 95% CIs were calculated using the Wilson score method.

^§^ Denominator excludes school districts that were not included in the surveillance area, calculated from National Center for Education Statistics enrollment counts of kindergarteners during the 2020–21 school year.

###  Case Ascertainment and Surveillance Case Definition

Surveillance was conducted using the same methods as in 2018 (*7*,*10*). All sites had access to health and education records in 2020 (*11*). Nine sites had data agreements in place with education sources covering 100% of their study areas, Missouri had agreements with education data sources covering 50.3%, and Georgia with education data sources covering 97.6% of its study area. Maryland, Utah, and Wisconsin had access to Individuals with Disabilities Education Act (IDEA) Part C early intervention data. Arizona, California, and Wisconsin also had access to state-funded services such as Medicaid (https://www.medicaid.gov/sites/default/files/2019-12/list-of-eligibility-groups.pdf) or disability services programs (*11*). Sites requested health records with *International Classification of Diseases, Ninth Revision* (ICD-9) or *International Classification of Diseases, Tenth Revision* (ICD-10) billing codes relevant to developmental disabilities from clinical sources and special education records with specific special education exceptionality codes from school sources to identify children with ASD.

Children aged 4 years in 2020 met the surveillance ASD case definition if they lived in the surveillance area at any time in 2020 and they received 1) a written ASD diagnostic statement (diagnosis) by a qualified professional, 2) a special education classification of ASD (eligibility), or 3) an ASD ICD-9 code between 299.00 and 299.99 or ICD-10 code in the F84 range (*10*). Children with an ICD code for Rett syndrome (F84.2) and no other indicators of ASD were excluded. Children met the suspected ASD case definition if they did not meet the criteria for ASD but records contained a qualified professional’s statement indicating a suspicion of ASD. Additional demographic information, developmental evaluations, special education plans, and IQ assessments were collected from records for these children.

### Additional Data Sources and Variable Definitions

The number of children aged 4 years living in each surveillance area was obtained from the U.S. Census vintage 2021 county-level single-year-of-age postcensal population estimates for 2020 (https://www.census.gov/programs-surveys/popest/technical-documentation/methodology.html). Surveillance areas at three sites (Arizona, California, and Minnesota) comprised subcounty school districts. For these sites, population estimates were adjusted using public school enrollment counts (https://nces.ed.gov/ccd/files.asp) of school districts included in surveillance areas. Full details are available (Supplementary Methods and Supplementary Table 1, https://stacks.cdc.gov/view/cdc/124396).

When race and ethnicity or sex was missing from records, birth certificate data were used if available. Co-occurring intellectual disability was defined as an IQ score of ≤70 on the most recent test or an examiner’s statement of intellectual disability in a developmental evaluation. Evaluation by age 36 months was calculated using the earliest recorded developmental evaluation (health or educational) for each child. Earliest identification was defined as the child’s first recorded ASD diagnosis or special education eligibility (identification ages for children with only an ICD code were not available).

### Analytic Methods

ASD prevalence was calculated as the number of children who met the ASD surveillance case definition per 1,000 children aged 4 years living in the surveillance area. Prevalence was calculated overall, by sex, and by race and ethnicity for American Indian or Alaska Native (AI/AN), Asian or Pacific Islander (A/PI), Black, White, two or more races (multiracial), and Hispanic children. (Persons of Hispanic origin might be of any race but are categorized as Hispanic; all racial groups are non-Hispanic). Children lacking information on sex (n = 4) or race (n = 94) were excluded from analyses stratified by those variables. Prevalence estimates with a relative SE ≥30% were suppressed because of limited statistical precision. Prevalence ratios were used to compare prevalence by sex, race and ethnicity, and suspected ASD from 2018 to 2020; prevalence ratios involving at least one suppressed estimate were likewise suppressed.

Cumulative incidence of ASD per 1,000 children was calculated by dividing the total number of children aged 4 or 8 years with an ASD diagnosis or eligibility at each month of age by the population denominator for children aged 4 years or 8 years in 2020. Diagnosis or eligibility by age 48 months was compared between children aged 4 years and 8 years in 2020 using risk ratios.

To assess the impact of COVID-19 on patterns of evaluation and identification, numbers of evaluations and ages of earliest identification were aggregated by calendar month for children aged 4 and 8 years in 2020. To compare the same age windows by calendar month, the numbers of evaluations and incidence of identification from 2012 (year 0) through 2016 (year 4) for children aged 8 years was subtracted from the same months during 2016 (year 0) through 2020 (year 4) for children aged 4 years.

The Wilson score method was used to calculate 95% CIs for prevalence, prevalence ratios, cumulative incidence, and risk ratios. Prevalence and risk ratios were considered significant when 95% CIs did not include 1.0. The male-to-female prevalence ratio among children aged 4 years was compared among sites, between years, and with the ratio among children aged 8 years (*11*) using the Mantel-Haenszel test of homogeneity. Chi-square tests or Fisher tests were used to compare differences in distributions between groups for analyses of co-occurring intellectual disability, evaluation by age 36 months, and having confirmed versus suspected ASD. Mantel-Haenszel and chi-square tests were considered significant when the p value was <0.05. R software (version 4.2.0; R Foundation) was used for data analysis and visualizations.

## Results

### Prevalence and Characteristics of Children Aged 4 Years with ASD

**FIGURE 1:**
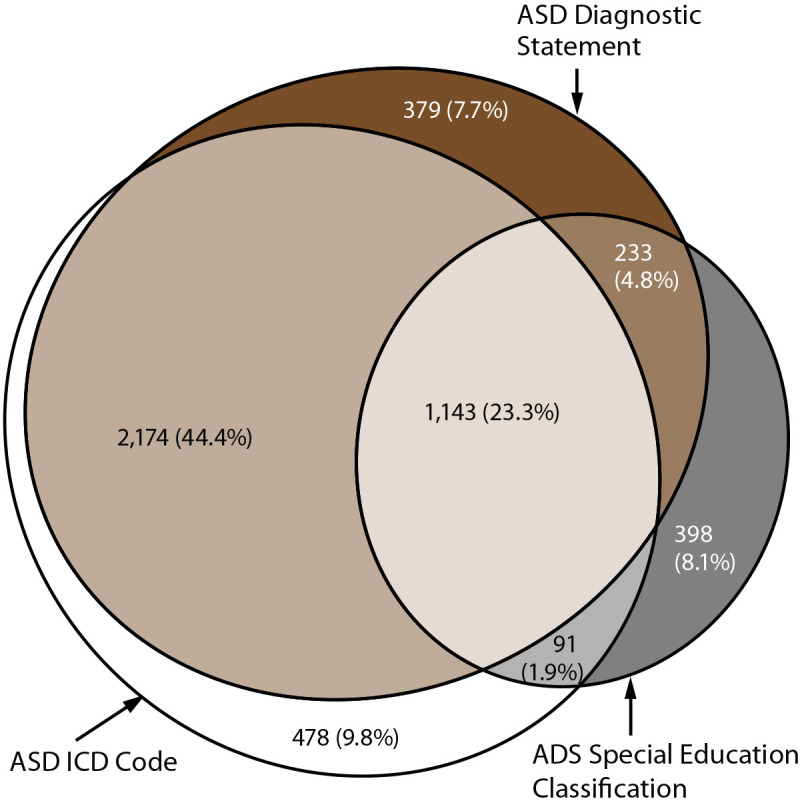
Euler diagram showing presence and overlap of case definition components among children aged 4 years with autism spectrum disorder (N = 4,896) — Autism and Developmental Disabilities Monitoring Network, 11 sites, United States, 2020 **Abbreviations:** ASD = autism spectrum disorder; ICD = *International Classification of Diseases*.

For 2020, prevalence ranged from 12.7 per 1,000 children aged 4 years in Utah to 46.4 in California (Table 1). ASD prevalence in the 11 sites combined was 21.5 per 1,000 (one in 46) children aged 4 years. Overall, 80.2% of children who met the ADDM Network case definition had a recorded ASD diagnosis (range = 54.1%–98.3%) (Figure 1) (Supplementary Figure 1, https://stacks.cdc.gov/view/cdc/124396), 38.1% had a recorded ASD eligibility (range = 9.3%–75.1%), and 79.4% had a recorded ASD ICD code (range = 22.0%–92.7%). A majority of children (74.4%) had more than one component of the case definition in their records and 23.3% had all three; 9.8% had an ASD ICD code only (Figure 1).

### Demographics

For 2020, prevalence was 32.3 per 1,000 boys aged 4 years, which was 3.1 times as high as among girls (10.4) (Table 2). The male-to-female ratio among children aged 4 years was similar across sites, but the ratio was lower than among children aged 8 years (*11*). Prevalence in 2020 among children aged 4 years was 24.3% higher among boys and 34.5% higher among girls than prevalence in 2018 (*7*), but the male-to-female ratios were similar between years. Among race and ethnicity groups, Hispanic children had the highest prevalence of ASD at 28.7 among children aged 4 years, followed by Black children (25.3), A/PI children (23.5), multiracial children (19.3), White children (16.3), and AI/AN children (11.1) (Table 3).

**TABLE 2:** Prevalence* of autism spectrum disorder among children aged 4 years, by sex^†^ — Autism and Developmental Disabilities Monitoring Network, 11 sites, United States, 2020

Site	Male ASD prevalence (95% CI)^§^	Female ASD prevalence (95% CI)	Male-to-female prevalence ratio (95% CI)^¶^
Arizona	24.2 (20.8–28.1)	6.9 (5.1–9.2)	3.5 (2.5–4.9)
Arkansas	24.9 (21.7–28.6)	6.9 (5.2–9.0)	3.6 (2.7–4.9)
California	71.7 (66.4–77.4)	20.5 (17.6–23.7)	3.5 (3.0–4.1)
Georgia	25.5 (22.7–28.6)	8.9 (7.3–10.9)	2.9 (2.3–3.6)
Maryland	26.4 (23.5–29.6)	7.3 (5.8–9.1)	3.6 (2.8–4.7)
Minnesota	29.7 (26.3–33.6)	7.3 (5.7–9.4)	4.1 (3.1–5.4)
Missouri	26.8 (24.1–29.8)	10.2 (8.6–12.2)	2.6 (2.1–3.2)
New Jersey	35.0 (31.5–38.9)	14.1 (11.9–16.7)	2.5 (2.0–3.0)
Tennessee	41.1 (37.8–44.5)	14.3 (12.4–16.4)	2.9 (2.4–3.4)
Utah	19.5 (17.3–22.1)	5.5 (4.3–7.0)	3.6 (2.7–4.7)
Wisconsin	33.4 (30.6–36.4)	11.2 (9.6–13.1)	3.0 (2.5–3.6)
**Total**	**32.3 (31.3–33.3)**	**10.4 (9.8–11.0)**	**3.1 (2.9–3.3)**

**Abbreviation:** ASD = autism spectrum disorder.

* Per 1,000 children aged 4 years.

^†^ Four children were missing sex information.

^§^ 95% CIs were calculated using the Wilson score method.

^¶^ Prevalence was significantly higher among males than females at all sites (95% CI excludes 1.0).

**TABLE 3:** Prevalence* of autism spectrum disorder among children aged 4 years, by race and ethnicity^†^ — Autism and Developmental Disabilities Monitoring Network, 11 sites, 2020

Site	ASD prevalence (95% CI)^§^					Prevalence ratio (95% CI)			
	White	Black	Hispanic	Asian/Pacific Islander	Multiracial	Black to White	Hispanic to White	Asian/Pacific Islander to White	Multiracial to White
Arizona	18.4 (15.0–22.5)	15.0 (8.9–25.0)	14.7 (12.0–18.2)	— ^¶^	—	0.8 (0.5–1.4)	0.8 (0.6–1.1)	—	—
Arkansas	13.6 (11.4–16.2)	22.4 (18.1–27.8)	17.6 (12.1–25.5)	—	15.8 (8.8–28.1)	1.6 (1.2–2.2)**	1.3 (0.9–1.9)	—	1.2 (0.6–2.1)
California	31.0 (26.2–36.6)	54.3 (42.6–68.8)	52.8 (48.1–57.9)	45.5 (37.1–55.6)	52.4 (41.7–65.7)	1.8 (1.3–2.3)**	1.7 (1.4–2.1)**	1.5 (1.1–1.9)**	1.7 (1.3–2.2)**
Georgia	8.1 (6.0–10.7)	21.9 (19.3–24.9)	16.1 (12.0–21.7)	17.4 (12.5–24.3)	20.8 (13.2–32.7)	2.7 (2.0–3.7)**	2.0 (1.3–3.0)**	2.2 (1.4–3.3)**	2.6 (1.5–4.4)**
Maryland	11.9 (10.0–14.1)	27.6 (23.3–32.5)	12.9 (8.9–18.6)	22.3 (16.5–30.2)	17.0 (11.3–25.7)	2.3 (1.8–3.0)**	1.1 (0.7–1.6)	1.9 (1.3–2.7)**	1.4 (0.9–2.2)
Minnesota	13.1 (10.7–16.1)	23.4 (19.0–28.7)	24.1 (17.8–32.4)	18.9 (14.4–25.0)	19.0 (12.4–28.8)	1.8 (1.3–2.4)**	1.8 (1.3–2.6)**	1.4 (1.0–2.0)**	1.4 (0.9–2.3)
Missouri	17.3 (15.4–19.5)	22.4 (18.8–26.5)	15.8 (10.1–24.5)	26.1 (17.6–38.5)	9.7 (5.6–16.9)	1.3 (1.0–1.6)**	0.9 (0.6–1.4)	1.5 (1.0–2.3)**	0.6 (0.3–1.0)
New Jersey	16.4 (13.3–20.3)	23.8 (20.1–28.1)	31.7 (27.8–36.1)	17.6 (11.4–27.0)	—	1.4 (1.1–1.9)**	1.9 (1.5–2.5)**	1.1 (0.7–1.7)	—
Tennessee	23.8 (21.5–26.3)	34.6 (29.6–40.3)	31.4 (26.3–37.5)	28.7 (19.7–41.8)	17.5 (12.0–25.5)	1.5 (1.2–1.7)**	1.3 (1.1–1.6)**	1.2 (0.8–1.8)	0.7 (0.5–1.1)
Utah	11.7 (10.2–13.5)	—	14.9 (11.9–18.5)	16.3 (10.4–25.2)	—	—	1.3 (1.0–1.6)**	1.4 (0.9–2.2)	—
Wisconsin	16.5 (14.6–18.6)	27.5 (23.4–32.2)	36.9 (32.0–42.6)	22.5 (16.3–31.0)	17.1 (11.6–25.1)	1.7 (1.4–2.0)**	2.2 (1.9–2.7)**	1.4 (1.0–1.9)**	1.0 (0.7–1.6)
**Total^††^**	**16.3 (15.5–17.0)**	**25.3 (23.9–26.8)**	**28.7 (27.2–30.3)**	**23.5 (21.2–26.1)**	**19.3 (17.0–21.9)**	1.6 (1.4–1.7)**	1.8 (1.6–1.9)**	1.4 (1.3–1.6)**	1.2 (1.0–1.4)**

* Per 1,000 children aged 4 years.

^†^ Persons of Hispanic origin might be of any race but are categorized as Hispanic; all racial groups are non-Hispanic. Excludes children of other or unknown race (n = 94).

^§^ 95% CIs were calculated using the Wilson score method.

^¶^ Dashes indicate suppressed estimate (relative SE ≥30% estimate or ratio involving at least one suppressed estimate).

** Significant prevalence ratio (95% CI excludes 1.0).

^††^ All site estimates for American Indian/Alaska Native (AI/AN) children were unstable (relative SE ≥30% estimate) and therefore not reported in the table. Overall AI/AN prevalence was 11.1 (95% CI = 6.2–19.7). Prevalence among AI/AN children was similar to White children (White-to-AI/AN prevalence ratio = 1.5; 95% CI = 0.8–2.6), but ASD prevalence was higher among Black (Black-to-AI/AN prevalence ratio = 2.3; 95% CI = 1.3–4.1), Hispanic (Hispanic-to-AI/AN prevalence ratio = 2.6; 95% CI = 1.4–4.6), A/PI (A/PI-to-AI/AN prevalence ratio = 2.1; 95% CI = 1.2–3.8), and multiracial (multiracial-to-AI/AN prevalence ratio = 1.7; 95% CI = 1.0–3.2) children.

Race and ethnicity patterns in prevalence were mostly consistent across sites. Compared with White children, ASD prevalence was higher among Black children at nine sites (overall 1.6 times as high), Hispanic children at seven sites (overall 1.8 times as high), A/PI children at six sites (overall 1.4 times as high), and multiracial children at two sites (overall 1.2 times as high) (Table 3). Prevalence among AI/AN children was similar to White children, but Black, Hispanic, A/PI, and multiracial children had an ASD prevalence 2.3, 2.6, 2.1, and 1.7 times as high as AI/AN children, respectively.

### Co-Occurring Intellectual Disability

Overall, 58.3% of children had information on intellectual ability available (range = 38.6% in Wisconsin to 89.8% in Arkansas) (Table 4). A/PI and multiracial children were more likely to have information on intellectual disability available (67% and 71.2% with information) than White (57.1%) and Black children (56.4%).

**TABLE 4:** Presence of co-occurring intellectual disability among children aged 4 years with autism spectrum disorder and available intellectual disability information, by site and selected characteristics — Autism and Developmental Disabilities Monitoring Network, 11 sites, 2020

Site/Characteristic	With intellectual disability information	With co-occurring intellectual disability
	No. (%)*	No. (%)^†^
Arizona	172 (82.3)	70 (40.7)
Arkansas	220 (89.8)	148 (67.3)
California	594 (76.5)	147 (24.7)
Georgia	273 (71.1)	155 (56.8)
Maryland	226 (64.2)	132 (58.4)
Minnesota	229 (75.1)	143 (62.4)
Missouri	185 (40.6)	93 (50.3)
New Jersey	228 (48.2)	135 (59.2)
Tennessee	327 (44.4)	182 (55.7)
Utah	148 (48.1)	64 (43.2)
Wisconsin	251 (38.6)	115 (45.8)
**Total**	**2,853 (58.3)**	**1,384 (48.5)**
**Sex^§^**		
Female	654 (56.4)	302 (46.2)
Male	2,198 (58.9)	1,081 (49.2)
**Race and ethnicity^¶^**		
Asian/Pacific Islander	231 (67.0)	114 (49.4)
Black	664 (56.4)	416 (62.7)
White	1,018 (57.1)	438 (43.0)
Multiracial	163 (71.2)	66 (40.5)
Hispanic	748 (59.5)	334 (44.7)

* Chi-square p values for significant comparisons for presence of intellectual disability information: White to Asian/Pacific Islander, White to multiracial, Black to Asian/Pacific Islander, Black to multiracial: p<0.01.

^†^ Chi-square p values for significant comparisons for presence of co-occurring intellectual disability: White to Black, Black to Hispanic, Black to Asian/Pacific Islander, and Black to multiracial: p<0.01.

^§^ One child with intellectual disability information was missing sex information

^¶^ Persons of Hispanic origin might be of any race but are categorized as Hispanic; all racial groups are non-Hispanic. Excludes children of other or unknown race; American Indian or Alaska Native not shown because of small numbers.

Among children with data on intellectual ability, 48.5% of children with ASD met the surveillance case definition of having co-occurring intellectual disability (range = 24.7% in California to 67.3% in Arkansas). The percentage was similar between boys (49.2%) and girls (46.2%) with ASD (Table 4), and among White, Hispanic, A/PI, and multiracial children with ASD (43%, 44.7%, 49.4%, and 40.5%, respectively). Black children with ASD had a higher percent of co-occurring intellectual disability than other groups at 62.7%. Fewer children had information about intellectual ability available at age 4 years (58.3%) compared with age 8 years (66.7%) in 2020 (*11*), but a higher percentage of children with information available met the surveillance case definition for intellectual disability among children aged 4 years (48.5%) compared with children aged 8 years (37.9%).

### First Evaluation

Among children with ASD with a developmental evaluation, 78% were evaluated by age 36 months, with variability across sites ranging from 66.7% in Tennessee to 87.8% in Arkansas (Table 5). Evaluation by age 36 months was similar by sex and by racial and ethnic groups except for being lower among Black (75.9%) compared with Hispanic (79.8%) children. Similar percentages of children with and without co-occurring intellectual disability were evaluated by age 36 months (82.4% and 85.1%), but the percentage was lower among children without intellectual ability information (68.5%).

**TABLE 5:** Percentage of children aged 4 years with autism spectrum disorder who had earliest recorded evaluation by age 36 months, by site and selected characteristics — Autism and Developmental Disabilities Monitoring Network, 11 sites, United States, 2020

Site/Characteristic	No. with evaluation	Evaluated by age 36 mos
		No. (%)*
Arizona	191	160 (83.8)
Arkansas	245	215 (87.8)
California	770	648 (84.2)
Georgia	341	269 (78.9)
Maryland	342	284 (83.0)
Minnesota	300	227 (75.7)
Missouri	456	343 (75.2)
New Jersey	472	377 (79.9)
Tennessee	649	433 (66.7)
Utah	291	221 (75.9)
Wisconsin	503	378 (75.1)
**Total**	**4,560**	**3,555 (78.0)**
**Sex^†^**		
Female	1,082	842 (77.8)
Male	3,477	2,712 (78.0)
**Race and ethnicity^§^**		
Asian/Pacific Islander	326	246 (75.5)
Black	1,087	825 (75.9)
White	1,653	1,307 (79.1)
Multiracial	218	174 (79.8)
Hispanic	1,182	943 (79.8)
**Co-occurring intellectual disability**		
Intellectual disability confirmed	1,372	1,131 (82.4)
No intellectual disability	1,452	1,235 (85.1)
Unknown	1,736	1,189 (68.5)

* Chi-square p values for significant comparisons: Black to Hispanic: p = 0.03, confirmed intellectual disability to unknown, and no intellectual disability to unknown: p<0.01.

^†^ One child with an evaluation was missing sex information.

^§^ Persons of Hispanic origin might be of any race but are categorized as Hispanic; all racial groups are non-Hispanic. Excludes children of other or unknown race; American Indian or Alaska Native not shown because of small numbers.

### Cumulative Incidence of ASD Diagnosis or Eligibility Compared with Children Aged 8 Years

**FIGURE 2:**
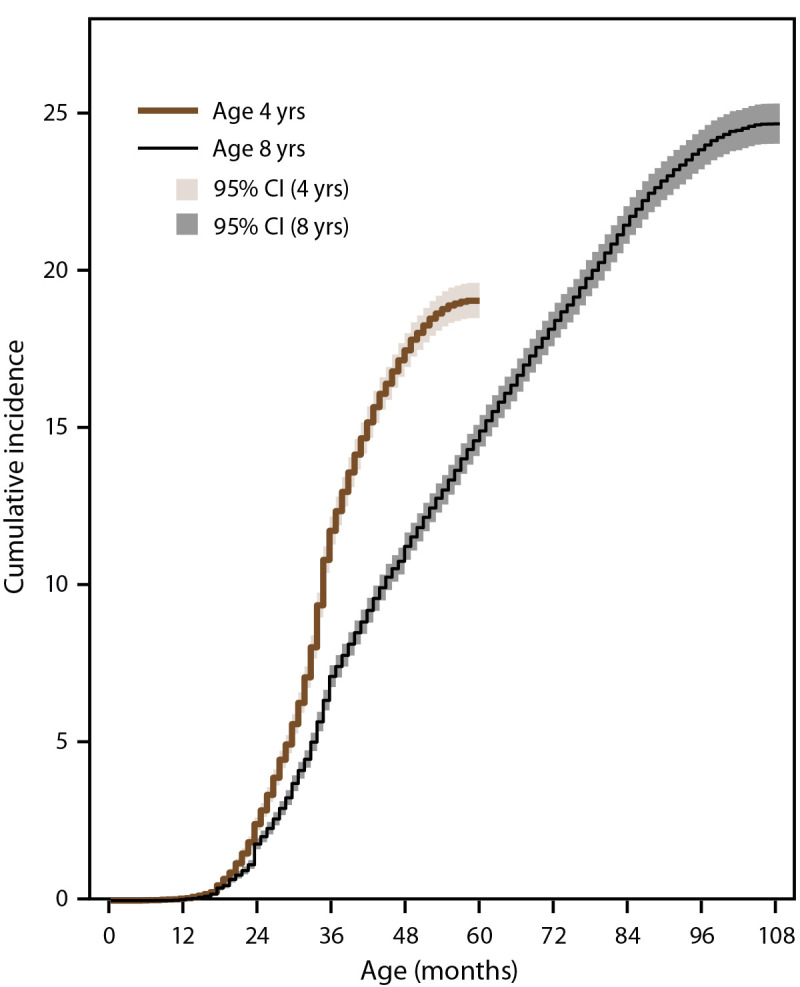
Cumulative incidence* of autism spectrum disorder diagnosis or autism special education classification (eligibility) among children aged 4 or 8 years, by month of age at identification — Autism and Developmental Disabilities Monitoring Network, 11 sites, United States, 2020 *Per 1,000 children aged 4 or 8 years.

Children aged 4 years had 1.6 times higher cumulative incidence of ASD diagnosis or eligibility by age 48 months than children aged 8 years in 2020 (17.5 per 1,000 children compared with 11.2) (Figure 2) (Supplementary Table 2, https://stacks.cdc.gov/view/cdc/124396). This pattern was consistent across sites, ranging from 1.3 times as high in New Jersey and Utah to 2.0 times as high in Tennessee (Supplementary Figure 2, https://stacks.cdc.gov/view/cdc/124396).

### Evaluation and Identification During COVID-19 Pandemic Onset

**FIGURE 3:**
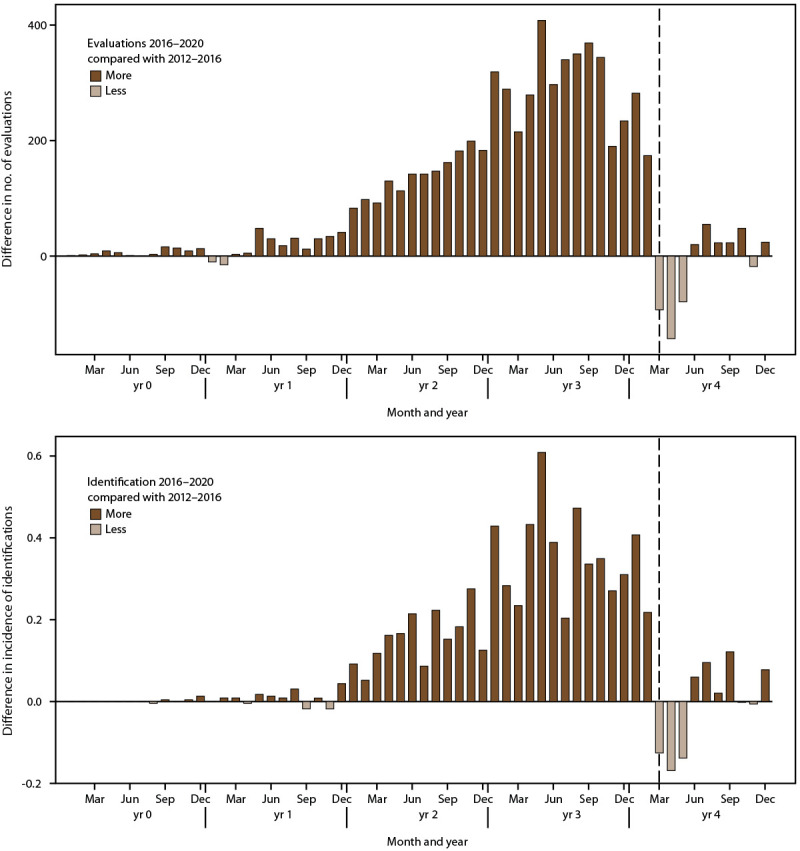
Difference in number of developmental evaluations and incidence* of autism spectrum disorder identification among children aged 4 years in 2020 during calendar years 2016–2020 and children aged 8 years in 2020 during calendar years 2012–2016, by month^†^— Autism and Developmental Disabilities Monitoring Network, 11 sites, United States *Per 1,000 children aged 4 years. ^†^ For children aged 4 years, year 0 to year 4 represents 2016–2020; for children aged 8 years, year 0 to year 4 represents 2012–2016. The dashed line shows the comparison at pandemic onset for children aged 4 years.

From 2016 through February 2020, children aged 4 years in 2020 had more evaluations and identifications than the cohort aged 8 years in 2020 had during 2012–2016. In the 6 months before the COVID-19 pandemic declaration in March 2020, there were 1,593 more evaluations and 1.89 more ASD identifications per 1,000 children aged 4 years than children aged 8 years received 4 years earlier (Supplementary Tables 3 and 4, https://stacks.cdc.gov/view/cdc/124396). After the COVID-19 pandemic declaration, this pattern reversed: In the 6 months after pandemic onset, there were 217 fewer evaluations and 0.26 fewer identifications per 1,000 children aged 4 years than children aged 8 years received 4 years earlier (Figure 3). Patterns of evaluations and ASD identification after pandemic onset varied by site, with certain sites (e.g., Georgia, Minnesota, and Utah) having sustained declines in evaluation and identification through the end of the year, but other sites (e.g, Missouri and Tennessee) showing resurgences closer to prepandemic levels (Supplementary Tables 3 and 4 and Supplementary Figures 3 and 4, https://stacks.cdc.gov/view/cdc/124396).

### Prevalence and Characteristics of Children Aged 4 Years with Suspected ASD

**FIGURE 4:**
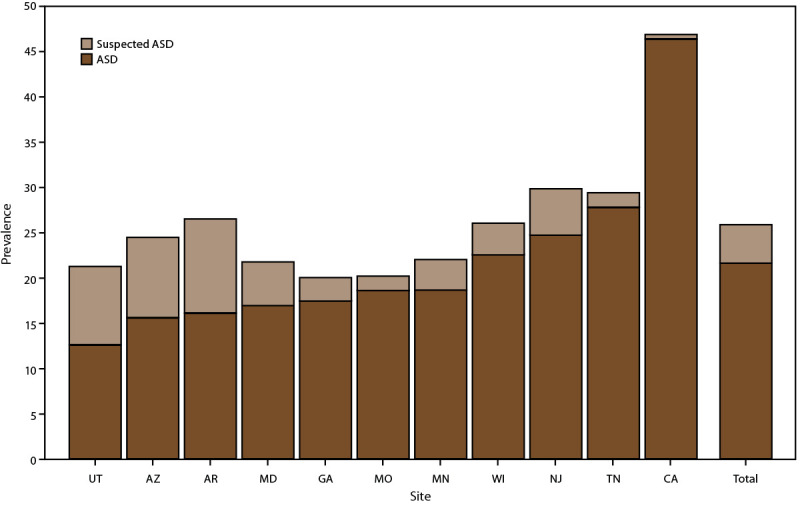
Prevalence* of autism spectrum disorder and suspected autism spectrum disorder among children aged 4 years — Autism and Developmental Disabilities Monitoring Network, 11 sites, United States, 2020 **Abbreviation:** Autism spectrum disorder. *Per 1,000 children aged 4 years.

**FIGURE 5:**
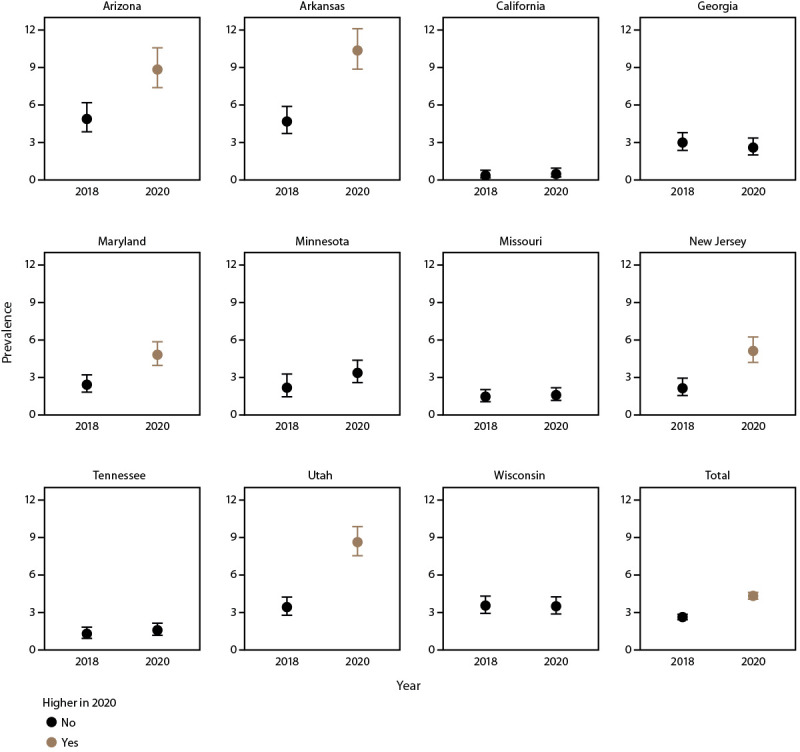
Prevalence* of suspected autism spectrum disorder among children aged 4 years — Autism and Developmental Disabilities Monitoring Network, 11 sites, United States, 2018 and 2020^†^ *Per 1,000 children aged 4 years. ^†^ Higher if 2020 to 2018 prevalence ratio 95% CI excludes 1.0.

Prevalence varied widely among sites in the prevalence of children with suspected ASD (those with a documented qualified professional’s statement indicating a suspicion of ASD but without a diagnosis, eligibility, or ICD code), from 0.5 per 1,000 children aged 4 years in California to 10.4 in Arkansas.), from 0.5 per 1,000 children aged 4 years in California to 10.4 in Arkansas (Figure 4) (Supplementary Table 5, https://stacks.cdc.gov/view/cdc/124396). Prevalence of suspected ASD increased from 2018 at five sites (Arizona, Arkansas, Maryland, New Jersey, and Utah) and was similar between years at other sites (*7*) (Figure 5). Children with ASD and children with suspected ASD were similar by sex, race and ethnicity, intellectual disability, and evaluation by age 36 months (Supplementary Table 6, https://stacks.cdc.gov/view/cdc/124396).

## Discussion

In 2020, ASD identification by age 48 months was higher among children aged 4 years than aged 8 years at all sites, indicating progress in early identification across the ADDM Network. Children aged 4 years had consistently more evaluations and higher identification from 2016 through February 2020 than children aged 8 had during 2012–2016. Despite the encouraging patterns, a large drop-off in progress among children aged 4 years was evident beginning with the pandemic onset in March 2020. Although every site experienced a period of lower evaluation and identification among children aged 4 years, certain sites showed movement toward recovery to prepandemic levels. This could reflect increased use of telehealth approaches that enabled remote evaluation and identification of children with ASD (*12*–*16*). Sustained decreases also could reflect extended remote learning through the end of 2020 in certain sites such as in Minnesota, where many children are identified in schools (Figure 1).

Suspected ASD was highest in the three states (Utah, Arizona, and Arkansas) with the lowest ASD prevalence, and two of the three states with the highest prevalence (California and Tennessee) had the lowest suspected ASD prevalence. Higher levels of suspected ASD could reflect community requirements for ASD diagnosis. For example, Arkansas code required agreement by three professionals in specified categories to receive an ASD diagnosis until the code was revised in 2021 to two “qualified professionals” (*17*), easing the requirements for identification. Suspected ASD prevalence in 2020 increased from 2018 at five sites and could be linked to challenges in evaluation and follow-up of children suspected of having ASD during the pandemic. Because earlier identification could lead to better outcomes, follow-up of children suspected but not confirmed of having ASD could help minimize delays in identification and intervention. The characteristics of children suspected of having ASD were similar to children with ASD, but these comparisons were among children seen for a developmental evaluation; disparities in receiving evaluations could still exist.

Prevalence among girls surpassed 10 per 1,000 children aged 4 years for the first time in ADDM Network surveillance (also observed among children aged 8 years for the first time) (*11*). A larger increase in prevalence from 2018 for girls compared with boys was observed; however, the male-to-female ratio was similar across years.

The higher prevalence of ASD among Black, Hispanic, and A/PI versus White children aged 4 years first reported in 2018 continued in 2020 and also was seen among children aged 8 years (*11*). This could reflect increased policy and community efforts to provide equitable services and access to health care for all children. The higher occurrence of ASD among these groups possibly suggests higher risk for ASD (e.g., perinatal or environmental factors) in populations that were previously masked by socioeconomic disparities in accessing ASD identification and treatment services. ASD is beginning to be identified more in lower-SES groups than previously (*7*,*11*); however, barriers to services still exist for lower-SES families of children with ASD (e.g., transportation, childcare, and job accommodations) (*18*–*26*).

Prevalence of ASD among children aged 4 years increased 26%, from 17.0 per 1,000 children in 2018 to 21.5 in 2020. However, prevalence varied widely across sites in 2020, with prevalence 265% higher in California than in Utah, the site with the lowest prevalence. This likely reflects community factors influencing early ASD identification. California continued to report high levels of early ASD identification, with higher prevalence at age 4 years than 8 years in 2020 (*11*). Local factors likely contribute to early identification at the California site, such as hundreds of pediatricians participating in the Get SET Early model (*27*) and a state-funded regional center that provides evaluations and service coordination for persons with disabilities and their families (https://www.dds.ca.gov). Variability in state-level procedures for early intervention also could contribute to differences in prevalence among sites. For example, in Maryland, early intervention is embedded within the school system, the health department, or shared between both (and other agencies such as social services). Such variability is likely seen across states and can potentially lead to different paths to accessing services.

The ADDM Network will continue tracking ASD identification, which might reveal lasting disruption to early identification and potential for longer-term impacts among children with ASD because of the COVID-19 pandemic. In addition, CDC’s Act Early Initiative promotes collaboration among early childhood programs in states and territories so children with autism or other delays and disabilities can be identified early and referred to appropriate services and supports (https://www.cdc.gov/ncbddd/actearly/about-initiative.html). In response to the pandemic, 43 state and territorial Act Early COVID-19 response teams, comprising representatives of multiple early childhood programs and state systems, were funded to support recovery; bolster early identification efforts; and strengthen resilience skills, behaviors, and resources of children, families, and communities. CDC’s “Learn the Signs. Act Early.” program also provides free developmental monitoring tools in multiple languages and the Milestone Tracker application to empower parents, educators, and health care providers to monitor children’s early development and address signs of developmental delay early (https://www.cdc.gov/ncbddd/actearly).

ADDM Network data for 2020 show differences across sites that suggest differences in early identification practices or policies. These data allow communities to explore barriers to, or success with, early identification of ASD for all children. The increases in identification are encouraging, but there is potential to improve early ASD identification in many ADDM Network communities, especially considering disruptions to developmental evaluation and services during the COVID-19 pandemic.

## Limitations

The findings in this report are subject to at least four limitations. First, surveillance area populations are not necessarily generalizable to the entire state or United States. Second, data quality is dependent on data availability and completeness of records at data sources. For example, demographic characteristics reflect what is documented in records; sex and race and ethnicity estimates could be higher or lower if administrative records are different from individual or family preferences. Third, sites or study areas could change between study years, which could complicate comparisons over time. The latter is mitigated by comparing ASD identification between children aged 4 and 8 years in the same study area during the surveillance period. Finally, the surveillance case definition of intellectual disability is not the same as a clinical diagnosis; IQ measurements in young children might lack stability and children might not ultimately receive a diagnosis of intellectual disability.

## Future Directions

Increasing access to additional data sources, such as Medicaid and IDEA Part C early intervention data, continues to be a goal of the ADDM Network’s early identification work. Data from upstream steps, including screening and referrals, could help identify inequities or barriers to services. Because the COVID-19 pandemic began part way through the surveillance year, future surveillance years could reveal the magnitude and persistence of COVID-19 pandemic effects on ASD identification in communities.

## Conclusion

Findings from the ADDM Network 2020 surveillance year include a higher prevalence of ASD among children aged 4 years than in 2018. ASD prevalence was higher among boys compared with girls, and among Hispanic, Black, and A/PI children compared with White and AI/AN children. Findings were similar to 2018 by presence of co-occurring intellectual disability and evaluation by age 36 months. The higher cumulative incidence of ASD identification by age 48 months among children aged 4 years compared with children aged 8 years indicates more early identification in the younger group. Children aged 4 years in 2020 received more evaluations and ASD identification before the March 2020 COVID-19 pandemic declaration than children aged 8 years received 4 years earlier. However, after pandemic onset, the pattern reversed, and fewer evaluations and identifications were observed among children aged 4 years. Identification of children with suspected ASD also increased at certain sites compared with 2018 data. Because of variability in practices across communities, and the unprecedented disruption to services caused by the COVID-19 pandemic, understanding factors to recover and improve equitable and timely access to early ASD identification and services could improve outcomes for children with ASD.
